# Necrotizing Fasciitis of the Breast Following a Core-Needle Biopsy

**DOI:** 10.7759/cureus.15786

**Published:** 2021-06-20

**Authors:** Taruna Singh, Abhay S Gaur, Gorrepati Rohith

**Affiliations:** 1 Surgery, University College of Medical Sciences, New Delhi, IND; 2 Urology, All India Institute of Medical Sciences, Bhubaneswar, Bhubaneswar, IND; 3 General Surgery, Jawaharlal Institute of Postgraduate Medical Education and Research, Puducherry, IND

**Keywords:** breast, group a β-haemolytic streptococci, core needle biopsy, necrotizing fasciitis, necrotizing fasciitis breast

## Abstract

Necrotizing fasciitis (NF) is an aggressive rapidly spreading infection of the skin and the subcutaneous tissue. Its occurrence in the breast is extremely rare especially after routine procedures like core-needle biopsy. We present the case of a 35-year-old non-lactating female who presented with swelling and a necrotic patch with pus discharge over her right breast following a core needle biopsy. She was immediately treated with aggressive debridement and culture-specific intravenous antibiotics following which she had an uneventful recovery. It is quintessential to diagnose the condition and initiate the treatment as early as possible to prevent the progression of the disease to sepsis and multi-organ dysfunction.

## Introduction

Necrotizing fasciitis (NF) is a highly aggressive and rapidly spreading superficial soft tissue infection. Mortality rates with this condition can reach as high as 73%, but a quick diagnosis and rapid intervention can duly reduce them [1]. Its occurrence in the breast is infrequent in patients with no risk factors, and only seven cases were reported in non-lactating women so far in the literature [2]. We present the case of a 35-year-old lady who presented with NF of the right breast due to group-A β-haemolytic streptococci infection following a core-needle biopsy and was successfully managed with immediate radical debridement and culture appropriate antibiotics.

## Case presentation

**Figure 1:**
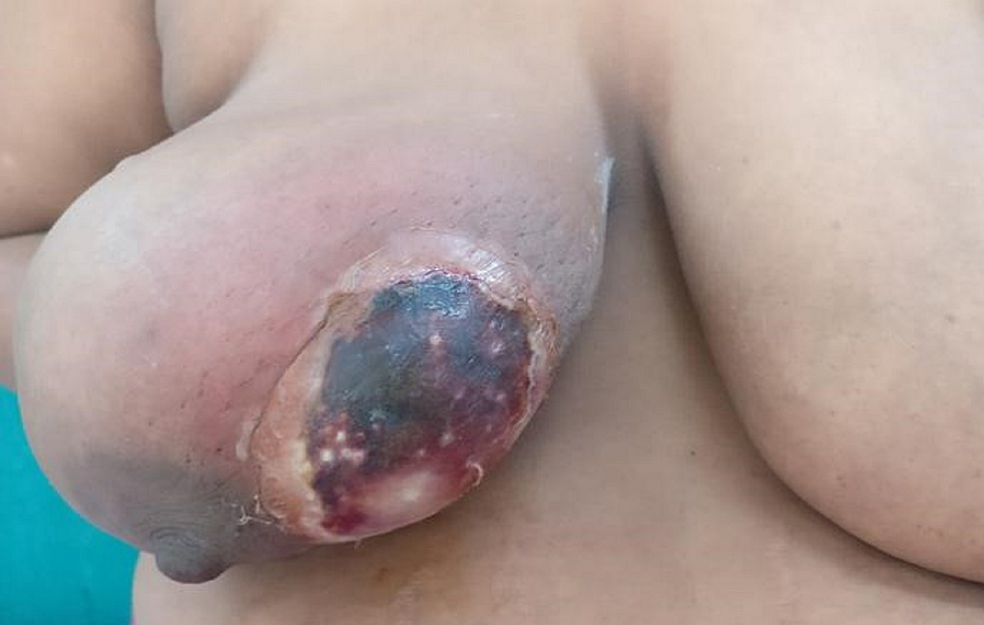
A 10 x 6 cm necrotic patch with surrounding erythema seen over the medial aspect of the right breast

A 35-year-old lady presented to the surgical outpatient department with complaints of a vague lump in the right breast for the past three months. The swelling was associated with an occasional pricking type of pain, which was non-radiating. The swelling was insidious in onset and slow-growing. She had no history of fever, trauma or nipple discharge. On examination, a vague 3x2 cm lump was palpable in the right breast upper inner quadrant with no associated erythema, tenderness or local rise in temperature. Ultrasonography of the breast was performed, which showed a hypoechoic cystic lesion of 3.4 x 2.1cm size with few focal areas of heterogenic solid parenchyma (BIRADS 4a). A core-needle biopsy of the lesion was performed under local anaesthesia to establish the diagnosis. No pre-biopsy antibiotic prophylaxis was given to the patient as per the institutional protocol. Four days later, the lady presented to the emergency with an increase in the size of the swelling at and surrounding the site of the procedure. She complained of severe pain and pus discharge from the location of the core-needle biopsy. She also had an intermittent high-grade fever for the past two days. She is a known diabetic with uncontrolled sugar levels at presentation. She had no other comorbidities. At presentation, her vitals were stable, and examination revealed a 10x6 cm swelling in the right breast upper inner quadrant with a necrotic patch over it and surrounding erythema (Figure 1).

There was a presence of foul-smelling discharge from the swelling. She also had mild right axillary lymphadenopathy. The opposite breast was normal. Her initial blood investigations revealed elevated total leukocyte counts (TLC - 19,000/mm^3^) and an elevated C-reactive protein (CRP - 55 mg/dL). Renal and liver function tests were within normal limits. Histopathological examination of the initial core-needle biopsy was suggestive of benign fibrocystic disease of the breast.

The patient was immediately taken for debridement under general anaesthesia in the emergency after obtaining informed consent. Intraoperatively, a 10x7 cm necrotic skin with an underlying 100 mL pus was mixed with necrotic material, which was debrided from swelling sparing nipple-areola complex. The debridement was extended until fresh bleed was found on the skin edges until we reached healthy breast tissue in the deeper layers. No definite mass/lump was palpable during the debridement at the area of the biopsy. She was transferred to an intensive care unit and immediately started on intravenous piperacillin-tazobactam 4.5 g every eight hours and clindamycin 600 mg every 12 hours. Her fever subsided on the following day, and her intraoperative cultures showed the growth of group-A β-haemolytic streptococci. Her blood glucose levels were controlled with insulin infusion. Following the antibiotic course, her TLC and CRP levels normalized. The wound was managed with serial dressings. After seven days of the antibiotic course, she was discharged in a stable condition with oral trimethoprim-sulfamethoxazole 400 mg twice daily for seven days. Her wound healed completely by secondary intention and was asymptomatic even at six months follow-up.

## Discussion

In the year 1952, NF was first described by Wilson as superficial necrosis of the skin and the subcutaneous tissue that spares the muscle [3]. Conditions such as advanced age, uncontrolled diabetes mellitus (DM), peripheral vascular disease, alcoholism, intravenous drug abuse, chronic renal failure and other immunosuppressive states were implicated as risk factors in the development of this fulminant disease. Our patient had uncontrolled DM at presentation, which was a significant risk factor in the development of the disease. NF is known to involve any part of the body. Still, it is particularly well described in the perineal region (Fournier's gangrene), abdominal wall (Meleney's gangrene) and the extremities where the occurrence of the disease is more common [4].

Two types of NF were previously described in the literature based on the causative organism. The type 1 NF is usually caused by a polymicrobial infection in which anaerobes, gram-positive and gram-negative, were implicated [5]. In type 2 NF, group-A streptococcus is implicated as the common etiological agent, and it usually occurs in patients with risk factors such as uncontrolled DM or obesity [6].

Diagnosis is mainly clinical, with the affected area showing signs of inflammation with reddish-purple to dusky blue discolouration of the overlying skin [7]. Rapid diagnosis of the condition and differentiating it from other aggressive conditions of the breast, such as inflammatory breast carcinoma, is of paramount importance [6]. NF of the breast shows a rapid progression culminating in septicaemia and multi-organ failure. Nevertheless, with prompt diagnosis and timely intervention, the mortality rates were known to reduce to as low as 10% [1,2].

Primary treatment modalities include early and aggressive surgical debridement and providing culture-specific antimicrobial therapy. The maintenance of haemodynamic and electrolyte balance is essential in patients who progress to generalized sepsis [8]. In severe cases, mastectomy is to be considered to prevent rapid deterioration and progression to multi-organ dysfunction as the condition carries high morbidity and mortality risk [2]. Nizami et al. and Fayman et al. described patients with a presentation similar to ours. They had to undergo a mastectomy due to the fulminant course of the disease leading to multi-organ dysfunction [2,7]. Conservative approaches pertaining to the conservative approach in the form of preserving breast tissue as much as possible can only be considered if the patient's general condition is stable and in the early stages of the disease with an indolent course. The defect can be reconstructed using standard techniques based on the site and the extent of the tissue loss but is usually delayed until the patient's complete recovery.

## Conclusions

Although rare in the breast, a high degree of clinical suspicion is necessary for the early diagnosis and management of NF, thereby preventing the need for mastectomy and preservation of most of the mammary tissue. Treating surgeons should be aware of other aggressive conditions such as inflammatory breast carcinoma, where the treatment varies significantly, and the prognosis is poor. A case of NF of the breast following a core-needle biopsy and its successful treatment with radical debridement alone was described.

## References

[1] Kaczynski J, Dillon M, Hilton J (2012). Breast necrotising fasciitis managed by partial mastectomy. BMJ Case Rep.

[2] Fayman K, Wang K, Curran R (2017). A case report of primary necrotising fasciitis of the breast: a rare but deadly entity requiring rapid surgical management. Int J Surg Case Rep.

[3] Wilson B (1952). Necrotizing fasciitis. Am Surg.

[4] Rohith G, Nelson T, Vinodhini P, Sahoo AK (2019). An unwonted case of Meleney’s like abdominal wall necrotizing soft tissue infection. Int Surg J.

[5] Flandrin A, Rouleau C, Azar CC, Dubon O, Giacalone PL (2009). First report of a necrotising fasciitis of the breast following a core needle biopsy. Breast J.

[6] Rajakannu M, Kate V, Ananthakrishnan N (2006). Necrotizing infection of the breast mimicking carcinoma. Breast J.

[7] Nizami S, Mohiuddin K, Mohsin-e-Azam Mohsin-e-Azam, Zafar H, Memon MA (2006). Necrotizing fasciitis of the breast. Breast J.

[8] Anaya DA, Dellinger EP (2007). Necrotizing soft-tissue infection: diagnosis and management. Clin Infect Dis.

